# Simulation-based training for enhancing psychiatric nurses’ knowledge and clinical competencies in communication and psychiatric history taking: a quasi-experimental study

**DOI:** 10.3389/frhs.2026.1847509

**Published:** 2026-07-22

**Authors:** Amal I. Khalil, Rawan F. Aldehani, Shroog A. Aljahdali, Waad A. Almehemadi

**Affiliations:** 1 King Abdullah International Medical Research Center, Jeddah, Saudi Arabia; 2 College of Nursing, King Saud Bin Abdulaziz University for Health Sciences, Jeddah, Saudi Arabia; 3 Ministry of National Guard Health Affairs (MNGHA), Jeddah, Saudi Arabia; 4 Faculty of Nursing, Menoufia University, Shebin Elkom City, Egypt

**Keywords:** clinical competence, communication, educational measurement, medical history taking, psychiatric nursing, quasi-experimental study, simulation training

## Abstract

**Background:**

Simulation-based education is a highly effective method for enhancing psychiatric nurses' communication and history-taking skills, effectively bridging the gap between theoretical knowledge and clinical practice in the relationally intensive field of mental health.

**Aim:**

This study aimed to assess the effectiveness of a simulation-based educational intervention in improving psychiatric nurses' communication and history-taking competencies through a quasi-experimental design.

**Methods:**

A quasi-experimental pre–post intervention study was conducted among psychiatric nurses working in selected psychiatric wards. Participants underwent a structured simulation-based training program delivered over four sessions, each lasting 45–60 min. The program incorporated standardized patient scenarios, role-play, guided reflection, and debriefing, all focused on therapeutic communication and systematic psychiatric history-taking. Data were collected using a validated knowledge assessment tool, an observational communication skills checklist, and a structured case history-taking performance tool. Descriptive and inferential statistics were employed to evaluate pre–post differences and intervention effects.

**Results:**

Participants were predominantly young to middle-aged nurses (mean age 37.4 ± 7.6 years), with balanced gender representation. Most held a bachelor's degree and had moderate clinical experience (≥5 years). Post-intervention findings revealed statistically significant improvements in nurses' knowledge, communication skills, and history-taking performance compared to baseline (*p* < 0.001). Large to very large effect sizes were observed across all domains, indicating clinically meaningful gains. Nurses showed marked enhancement in the use of open-ended questions, empathic responses, structured data collection, and integration of psychosocial and clinical information during both simulated and clinical encounters.

**Conclusion:**

Simulation-based training is an effective and impactful educational strategy for reframing psychiatric nurse–patient interactions as intentional, therapeutic processes. The intervention significantly improved psychiatric nurses' communication and history-taking competencies, supporting the integration of simulation-based learning into psychiatric nursing education and continuing professional development programs.

## Introduction

Effective communication and accurate history-taking are fundamental competencies in psychiatric nursing. These skills are essential for establishing therapeutic relationships, conducting comprehensive mental health assessments, promoting patient engagement, and supporting treatment adherence and recovery. Therapeutic communication extends beyond information exchange and includes empathy, active listening, emotional support, and the creation of a psychologically safe environment that encourages patients to share sensitive experiences and concerns (1, 2). Similarly, comprehensive psychiatric history-taking provides critical information regarding psychosocial stressors, trauma exposure, substance use, medication adherence, suicidal ideation, and family psychiatric history, all of which contribute to accurate diagnosis and individualized treatment planning (3).

Despite their importance, deficiencies in communication and psychiatric interviewing skills are common among psychiatric nurses. Previous studies have reported a reliance on task-focused and closed-ended questioning styles, limited use of empathetic responses, and difficulties in discussing sensitive issues such as suicide, trauma, self-harm, and sexual health (4–6). Although communication and interviewing skills are included in nursing curricula, traditional educational approaches often emphasize theoretical instruction, with limited opportunities for experiential learning and clinical application. Consequently, many nurses enter psychiatric practice with insufficient confidence and competence to conduct therapeutic interviews independently (7).

Simulation-based training has emerged as an evidence-based educational strategy for improving communication, clinical competence, critical thinking, and professional confidence among healthcare workers (8). Through standardized patients, realistic clinical scenarios, structured feedback, and guided debriefing, simulation provides a safe environment for practicing therapeutic communication and psychiatric interviewing skills while facilitating the transfer of theoretical knowledge into clinical practice (9, 10). Previous studies have demonstrated significant improvements in communication skills, empathy, psychiatric assessment, self-confidence, clinical decision-making, and interview performance following simulation-based psychiatric education interventions (11–14).

However, existing evidence has primarily focused on communication skills, self-confidence, or undergraduate nursing students, with limited attention to practicing psychiatric nurses and psychiatric history-taking competencies (15). Furthermore, few studies have examined the combined effects of simulation-based training on both therapeutic communication and psychiatric history-taking skills in psychiatric nursing practice (16). Therefore, this study aimed to examine the effectiveness of a simulation-based training program in strengthening psychiatric nurses' knowledge, therapeutic communication competencies, and psychiatric history-taking performance, which are fundamental to comprehensive psychiatric patient assessment.

In the field of psychiatric nursing, the ability to communicate therapeutically and gather comprehensive patient histories is crucial for accurate assessments, building therapeutic relationships, and achieving favorable mental health outcomes. Despite their importance in psychiatric practice, research shows that psychiatric nurses often exhibit gaps and inconsistencies in these skills, especially in complex clinical settings with high patient acuity, time constraints, and organizational challenges (17). These shortcomings can undermine the accuracy of assessments, restrict patient disclosure, weaken care engagement, and negatively impact recovery paths. This study is significant because it focuses on redefining psychiatric nurse–patient interactions through simulation-based training, treating communication and history-taking as deliberate, structured, and therapeutic processes rather than routine tasks. By assessing a simulation-based educational intervention through a quasi-experimental design, this study addresses a critical gap in psychiatric nursing education and ongoing professional development, where experiential and reflective learning methods are underutilized (18). The study's findings could guide evidence-based educational strategies, support competency-based workforce development, and improve the quality of psychiatric nursing practice by fostering patient-centered, empathetic, and clinically effective nurse–patient interactions, ultimately leading to better patient outcomes and enhanced mental health care delivery (19, 20).

**Figure 1:**
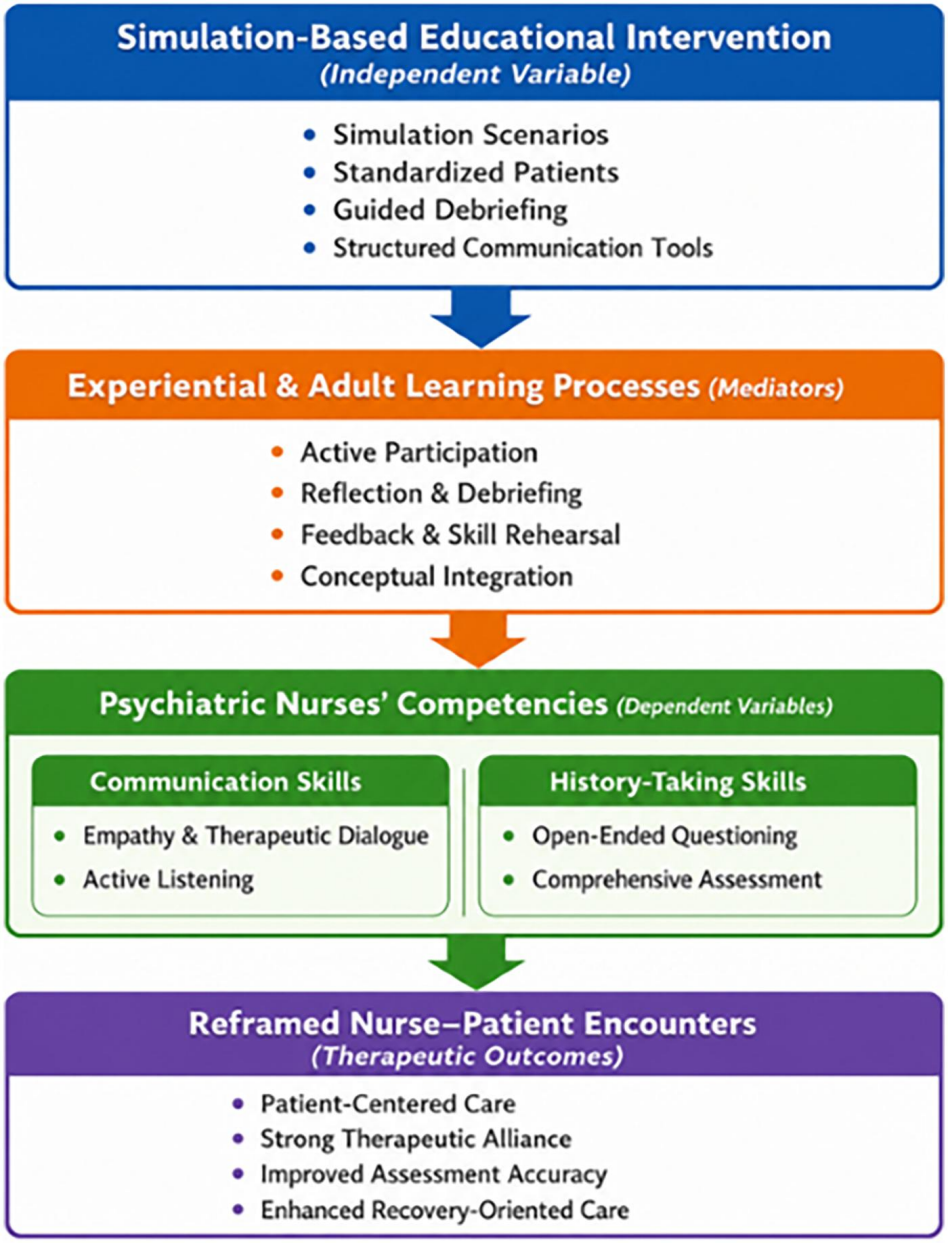
Application of the theoretical framework to the study variables.

The integrated theoretical framework presented in Figure 1 of this research study combines Peplau's Interpersonal Relations Theory, Adult Learning Theory, and Experiential Learning Theory to explain the positive impact of simulation-based training on communication and history taking among psychiatric nurses. Peplau's Interpersonal Relations Theory provides the foundation from which to perform an appropriate psychiatric assessment and to provide adequate care within a therapeutic relationship, by utilizing effective communication and systematic history taking (21).

The Adult Learning Theory has provided an educational design for the intervention that is based on the fact that adults are self-directed learners who learn best from relevant experiential experiences through their practice. The use of simulation-based training would provide the necessary practice experience and facilitate the development and maintenance of engagement and motivation in the acquisition of skills for practicing nurses (22).

According to the Experiential Learning Theory, the learning that occurs through simulation will be translated to practice as a result of undergoing a process of repeated structured reflection, debriefing, and repeated skill application. This will enhance the development of reflective practice and communication and history taking in clinical practice (23).

Collaboration between these three theories provides a common framework that links the practice of psychiatric nursing and simulation-based learning and supports the research hypothesis that simulation training will enhance the competency of psychiatric nurses in communication and history taking.

Experiential learning principles underlie simulation-based education, where experiential learning is built on concepts of active participation, reflection and feedback that lead to skill development and professional advancement. Simulation gives nurses the opportunity to connect their theoretical knowledge to their clinical practice through practical and realistic clinical situations, thus enabling them to develop the essential competencies needed to work effectively as psychiatric nurses. Though vitamin-based simulation research has been expanding exponentially, there is a lack of studies indicating the effectiveness of simulation training programs in developing knowledge, communication and psychiatric history-taking skill sets among nurses who work with clients in mental health environments. Filling this gap is critical since these skill sets are necessary to create accurate assessments, therapeutic relationships and to provide safe, effective care for clients. The purpose of this study is to evaluate the effectiveness of a simulation-based educational program to improve psychiatric nurses' knowledge, communication and psychiatric history-taking skills. It is hypothesized that the nurses who participate in the program will exhibit statistically significant gains in the outcomes being measured following the intervention.

## Materials and methods

### Study design

A quantitative quasi-experimental (pre/post-one-group) design was utilized to meet the study's goals. While randomized controlled trials are often seen as the gold standard, a quasi-experimental design was chosen as a practical and ethical option suitable for applied healthcare environments, balancing scientific rigor with practical feasibility. In addition, this design was employed because of practical and administrative considerations within the clinical setting, including staffing constraints and the difficulty of withholding educational training from another comparable group of psychiatric nurses during the study period. This limitation has been acknowledged in the manuscript.

### Study area/setting

This study was conducted at Erada and Mental Health Complex in Jeddah, Saudi Arabia, a specialized psychiatric hospital operated by the Ministry of Health. The hospital serves as a major referral center for mental health services in the Western Region of Saudi Arabia and provides comprehensive psychiatric care through multidisciplinary medical, psychological, social, rehabilitative, and emergency services. The facility has a total licensed capacity of 150 beds.

The hospital includes inpatient psychiatric wards, outpatient psychiatric clinics for adults and children, psychiatric emergency services, rehabilitation programs, and acute care units for patients experiencing psychiatric crises and severe mental health conditions. The inpatient services comprise three acute psychiatric wards for adult male patients and two acute psychiatric wards for adult female patients, with each acute ward accommodating 18 beds. In addition, the hospital includes three subacute psychiatric wards with a combined capacity of 45 beds. The separation of male and female wards is consistent with Saudi healthcare regulations and cultural norms, whereby female nurses predominantly provide care to female patients and male nurses provide care to male patients.

At the time of the study, the hospital employed 114 psychiatric registered nurses distributed across inpatient wards, emergency services, and outpatient clinics. Nursing services were delivered through a three-shift rotation system (morning, evening, and night shifts) to ensure continuous patient care. Staffing levels were highest during the morning shift to support patient assessments, treatment activities, multidisciplinary rounds, and rehabilitation programs. On average, each acute psychiatric ward was staffed by approximately seven nurses during the morning shift, while each subacute psychiatric ward was staffed by approximately twelve nurses. The psychiatric emergency department employed approximately seven nurses, and outpatient psychiatric services employed ten nurses, with one nurse assigned to each outpatient clinic. Staffing levels during evening and night shifts were adjusted according to patient acuity, service demands, and hospital staffing policies.

### Study subjects

The study included 75 psychiatric nurses working during the morning shift at Erada and Mental Health Complex. Morning-shift nurses were specifically selected because therapeutic communication, psychiatric interviewing, patient assessment, admissions, multidisciplinary rounds, and history-taking activities are predominantly conducted during morning working hours. In addition, selecting morning-shift nurses enhanced the feasibility and consistency of implementing the simulation-based intervention sessions, observations, and debriefing activities within the available staffing and research resources. Second, the study did not include all nurses because of feasibility considerations, staffing logistics, and the operational demands of the psychiatric hospital. Conducting repeated simulation sessions, observations, debriefing activities, and pre–post assessments with the entire nursing workforce was not practically feasible within the available study period and resources. Therefore, a purposive sample of eligible nurses was recruited.

Eligible participants were nurses working in psychiatric inpatient, outpatient, emergency, and acute psychiatric care settings The term “acute psychiatric setting” refers to units managing patients experiencing acute psychiatric symptoms and crises, rather than being limited exclusively to outpatient psychiatric emergencies Regarding the educational level, the inclusion criteria initially specified nurses holding at least a bachelor's degree in nursing; however, some participants had additional postgraduate qualifications, which explains the variation in educational levels reported in the results section.

### Sample size estimation

The sample size was calculated using the Rao-Soft sample size calculation program https://www.raosoft.com/samplesize.html based on the total population of 75 nurses at the time of data collection. The calculation included a 5% margin of error, a 95% confidence level, and a response distribution of 50%, leading to a recommended sample size of 62 nurses. The sample size and margin of error were determined using the following formulas:

In terms of the numbers, you selected above, the sample size *n* and margin of error E are given by

$$\begin{array}{rl} & \begin{array}{c} x=Z(c/100)2r(100-r)\\ n=Nx/((N-1)\;E2\;+\;x) \end{array}\\ & E=\mathrm{Sqrt}[\left(N-n)\;x/n(N-1\right)] \end{array}$$

Where *N* is the population size, *r* is the fraction of responses that you are interested in, and Z(c/100) is the critical value for the confidence level.

### Sampling technique

A non-probability convenience sampling method was employed to efficiently and cost-effectively recruit a representative cohort of nurses, aligning well with the study's objectives. Data collection was conducted in 2024, targeting nurses working morning shifts in inpatient, outpatient, and emergency units. The nursing department at Erada and the Mental Health Complex provided initial approval for the study. Staff distributed pre- and post-intervention surveys in both paper and digital formats, enabling researchers to assess improvements in communication skills and history-taking abilities.

### Tools of the study

An English questionnaire gathered data to meet the study's aims. It included three main parts:

#### Demographic characteristics

This part asked for details like age, gender, marital status, years of experience, education level, and attendance at workshops on communication and history-taking. It covered other key demographics, too.

#### Knowledge assessment scale

The Knowledge Assessment Scale, created by Khalil (2025) (24), was formulated after an extensive examination of the pertinent literature and previous research. For the current study, the researchers modified the scale to better fit the goals and content of a simulation-based educational program. This tool is intended to evaluate nurses' knowledge of critical areas of psychiatric nursing practice. It comprises eight primary categories, each with specific subitems. The domains included Understanding the Importance of Effective Communication in Psychiatric Nursing (5 items), Building a Therapeutic Nurse–Patient Relationship (5 items), Effective Communication Skills (3 items), Psychiatric History-Taking (7 items), Managing Challenging Situations (5 items), Cultural Competence in Psychiatric Nursing (2 items), Role-Playing and Simulation Exercises (2 items), and Interdisciplinary Collaboration (2 items). Responses were gauged using a 3-point Likert scale, where participants expressed their level of agreement with each statement as Agree (3 points), Unsure (2 points), or Disagree (1 point).

The total score ranged from 30 to 90, with higher scores indicating greater knowledge. Scores were classified as follows: 30–45 (weak knowledge), 46–61 (fair knowledge), 62–77 (solid knowledge), and 78–90 (strong knowledge).

#### Validity and reliability

The content validity of the Knowledge Assessment Scale was confirmed through expert evaluation using the Content Validity Index (CVI). A group of specialists in psychiatric and mental health nursing and nursing education assessed the items for relevance, clarity, and representativeness. First, regarding the psychiatric nursing knowledge questionnaire, the Content Validity Ratio (CVR) and Content Validity Index (CVI) were established during the expert validation phase. The tool was reviewed by a panel of psychiatric nursing and mental health experts to evaluate item relevance, clarity, and representativeness. The questionnaire demonstrated excellent content validity, with a mean CVR of 0.89 and an overall Scale-Level Content Validity Index (S-CVI) of 0.94, indicating strong agreement among experts regarding the appropriateness and adequacy of the instrument items.

**Construct validity** was supported by the theoretical framework underlying the scale, which was organized into eight competency-based domains reflecting the essential areas of psychiatric nursing practice. The scale's structure aligns with established educational and clinical competencies and can detect changes in knowledge levels following educational or simulation-based interventions, thereby supporting its ability to measure the intended constructs.

**Face validity** was assessed through pilot testing with psychiatric nurses, who reported that the items were clear, relevant, and appropriate for their clinical practice context.

**In terms of reliability, internal consistency** was evaluated using Cronbach's alpha coefficient, which yielded a value of 0.86, indicating good reliability of the scale. This demonstrates that the scale items are consistent in measuring the overall construct of psychiatric nursing knowledge.

### Communication skills checklist

The Communication Skills Checklist was used to assess nurses' proficiency in therapeutic communication during psychiatric patient interviews. The checklist consists of 35 items adapted from Chauhan et al. (25), with an additional 10 items incorporated from the Manual of Psychiatric Clinical Competencies developed by Khalil (26). These additional items assess essential introductory and attending behaviors, including greeting the patient, self-introduction, and the use of attending behaviors.

The final checklist is organized into four interview phases: setting up the interview, opening the interview, conducting the interview, and closing the interview. The checklist comprises nine domains: attending behavior (6 items), rapport building (9 items), introduction (3 items), clarity (4 items), attention (8 items), responsiveness (5 items), empathy (3 items), support (5 items), and summarization (2 items), with a total possible score ranging from 0 to 45.

### Scoring and competency classification

Each item is scored dichotomously (1 = achieved, 0 = not achieved). Based on the total score, nurses' communication skills were categorized into three competency levels:

- Competent: ≥75% of the total score (34–45)
- Partially competent: 50%–74% of the total score (23–33)
- Not competent: <50% of the total score (0–22). Higher scores indicate better communication competency.

### Validity and reliability

The checklist underwent translation and back-translation procedures to ensure linguistic accuracy and conceptual equivalence between the Arabic and English versions. Content validity was established through review by five experts in psychiatric nursing and nursing education who evaluated the relevance, clarity, and applicability of the checklist items. Prior to the main study, the checklist was pilot tested with 10 psychiatric nurses to assess feasibility and clarity.

Because the checklist is observational in nature, inter-rater reliability was evaluated during the pilot phase. Two trained evaluators independently assessed a subset of simulation performances using the checklist, and Cohen's kappa coefficient demonstrated acceptable inter-rater agreement, supporting the consistency of observational scoring. Internal consistency reliability of the Arabic version was also assessed using Cronbach's alpha coefficient, which yielded a value of **0.86**, indicating high reliability and internal consistency.

### History-taking competency checklist

The History-Taking Competency Checklist was used to evaluate nurses' ability to obtain a comprehensive psychiatric history. The competency areas included patient demographic data, presenting complaints, current and previous medications, past psychiatric and medical history, family history, personal history, premorbid personality, substance use history, and forensic history. The checklist comprises 19 observational items developed by Khalil (26) and approved by the Clinical Skills Review Committee at the College of Nursing, King Saud bin Abdulaziz University for Health Sciences. The total possible score ranges from 0 to 58.

### Scoring and competency classification

Each item is scored dichotomously (1 = achieved, 0 = not achieved). Based on the total score, history-taking competency was classified into three levels:

- Competent: ≥75% of the total score (44–58)
- Partially competent: 50%–74% of the total score (29–43)
- Not competent: <50% of the total score (0–28). Higher scores indicate better psychiatric history-taking competency.

### Validity and reliability

The checklist underwent translation and back-translation procedures to ensure accuracy and consistency of the Arabic version. Content validity was established through expert review by specialists in psychiatric nursing and nursing education. The checklist was pilot tested with 10 psychiatric nurses to evaluate clarity, feasibility, and applicability within the psychiatric clinical simulation setting.

As the checklist involved observational assessment, inter-rater reliability was established through independent scoring by two trained evaluators during the pilot phase. Cohen's kappa coefficient demonstrated acceptable agreement between raters, supporting the reliability and consistency of the observational evaluations. Internal consistency reliability was assessed using Cronbach's alpha coefficient, which yielded a value of 0.83, indicating good reliability of the checklist.

### Research implementation phase

The research team executed the simulation-based educational program in assigned psychiatric units and training areas. Participants were divided into small groups, and the intervention was implemented through four in-person sessions (45–60 min each) that included short interactive lectures, group discussions, simulation activities, and organized debriefing. The simulation scenarios covered prevalent psychiatric disorders, such as depression, schizophrenia, bipolar disorder, and substance-induced psychosis, emphasizing therapeutic communication and comprehensive psychiatric history-taking.

**Figure 2:**
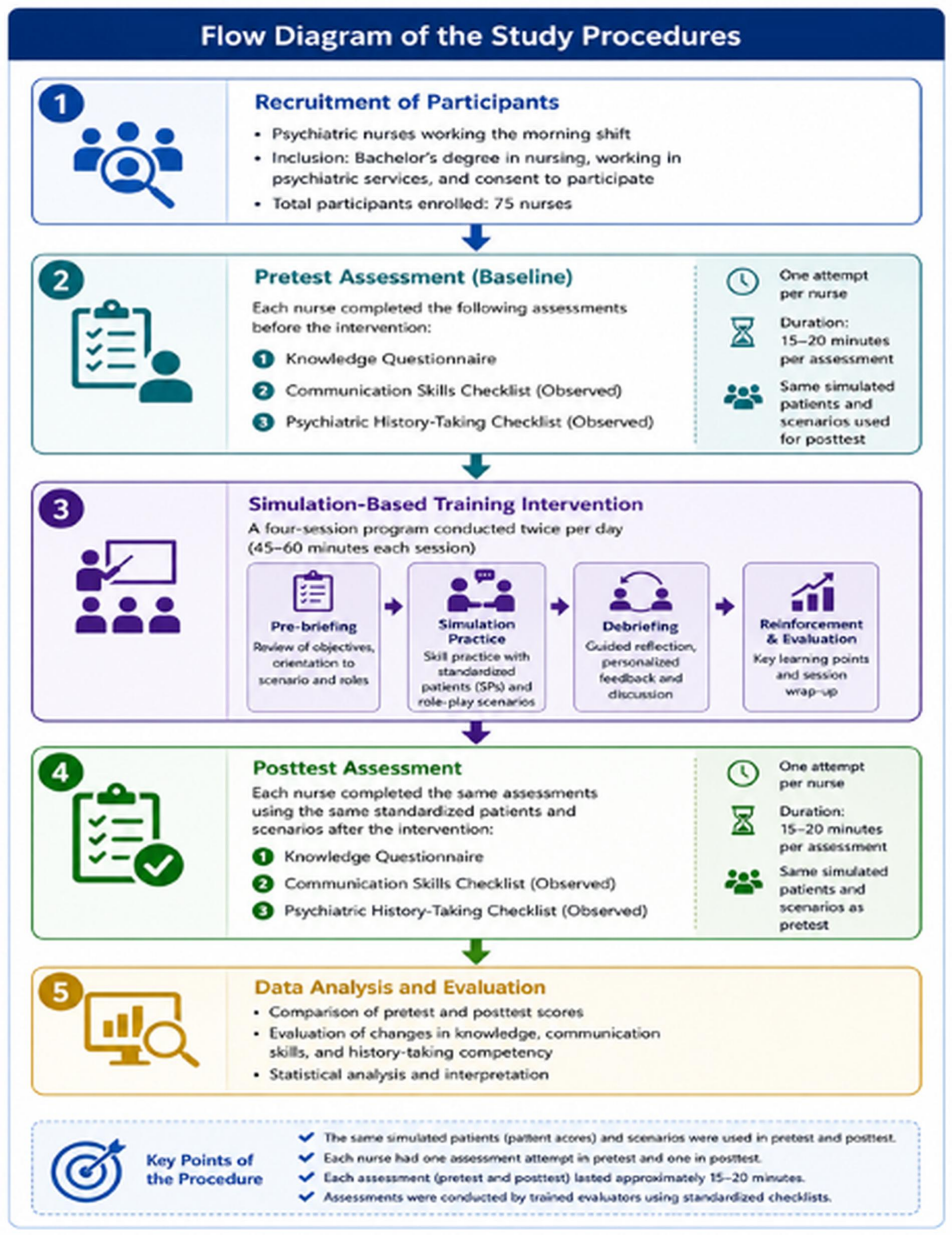
Summarizes the simulation-based program and data collection procedure.

To maintain consistency, identical standardized patient scenarios and trained simulated patients were utilized in both pre- and post-intervention evaluations. Every participant carried out a pretest and a posttest simulation evaluation, both of which were assessed using standardized competency checklists. Prompt feedback and directed reflection were given after every simulation session. More information about the intervention elements, execution process, and educational framework can be found in Table 1 and Figure 2.

**Table 1:** Simulation-based training program for psychiatric nurses: keys summary:.

Key Component	Description
Program aim	To enhance psychiatric nurses’ therapeutic communication and systematic psychiatric history-taking through immersive simulation-based learning grounded in theory-driven practice.
Theoretical framework	Adult Learning Theory (learner-centered, self-directed learning) (21); Experiential Learning Theory (concrete experience, reflection, application) (22); Peplau’s Interpersonal Relations Theory (therapeutic nurse–patient relationship) (23).
Design & duration	Four simulation-based sessions were delivered face-to-face; each session lasted 60–90 min.
Participants	Small groups of 6–8 psychiatric nurses to maximize engagement, individualized practice, and feedback.
Setting	Hospital inpatient wards or designated psychiatric training rooms.
Facilitators	Experienced psychiatric nurse educators trained in simulation facilitation and structured debriefing with coauthors.
Pre-briefing	Orientation to the simulation environment, clarification of learning objectives, establishment of psychological safety, and review of communication and history-taking frameworks.
Simulation scenarios	Realistic nurse–patient encounters using standardized patients or scenario enactments representing common psychiatric conditions (e.g., depression, schizophrenia, bipolar disorder, and substance-induced psychosis).
Core skills targeted	Therapeutic communication (empathy, active listening, emotional sensitivity) and structured psychiatric history-taking (psychosocial assessment, substance use, trauma, suicidality, medication adherence).
Debriefing & reflection	Structured facilitator-led debriefing following each scenario, emphasizing reflective practice, performance feedback, and application to clinical care.
Skill reinforcement	Progressive scenario complexity, repeated practice, peer observation, and guided feedback to consolidate learning.
Teaching methods	Simulation-based practice supported by brief interactive lectures, small-group discussions, instructional videos, PowerPoint presentations, and informational booklets.
Evaluation	Competency-based checklists are used for formative and summative assessment; pre- and post-program performance is reviewed with individualized feedback.
Implementation strategy	Four sessions delivered daily across psychiatric wards; two groups trained per day to optimize participation and learning engagement.
Expected outcome	Improved communication proficiency, enhanced psychiatric history-taking accuracy, and strengthened therapeutic nurse–patient relationships in clinical practice.

### Data management and analysis plan

Data management, verification, and statistical analyses were conducted under the supervision of a qualified biostatistician to ensure the accuracy and appropriateness of the statistical procedures. The data management and analysis for this study were conducted using the Statistical Package for the Social Sciences (SPSS), Version 25. Descriptive statistics were employed to summarize the study variables. Categorical variables, such as gender, education level, years of experience, and job position, are presented in terms of frequencies and percentages. For continuous variables, such as age, means and standard deviations were calculated. Inferential analyses included paired t-tests to compare participants' pre-test and post-test scores in communication and competence in history taking. Pearson's correlation coefficient (r) or Chi-square tests, where applicable, were utilized to assess the correlations among sociodemographic variables, the effectiveness of the educational program, and perceived barriers to completing the educational program. The level of statistical significance was established at *p* < 0.05.

## Results

Table 2 outlines the participants' demographic and professional profiles. Almost half of the nurses (46.7%) were between 25 and 35 years old, with 36.0% aged 36 to 46, and 17.3% over 46 years, resulting in an average age of 37.4 ± 7.6 years. The sample consisted of 53.3% females and 46.7% males, and 70.7% of the respondents were married. The majority (61.3%) possessed a bachelor's degree, followed by 18.7% with a diploma, 16.0% with a master's degree, and 4.0% with a doctorate. Participants were nearly evenly split between inpatient (52.0%) and outpatient (48.0%) settings. In terms of professional experience, 56.0% had worked for 5–9 years, while 44.0% had over 10 years. The most frequently reported previous training was in history-taking (42.7%) and therapeutic communication (38.7%), with only 10.7% having prior training in patient care issues.

**Table 2:** Distribution of the demographic characteristics of the study sample *N*=75.

Variables	*n*	%
Age (Years)		
25–35	35	46.7
36–46	27	36.0
>46	13	17.3
Mean ± SD	37.4 ± 7.6	
Gender		
Female	40	53.3
Male	35	46.7
Marital status		
Married	53	70.7
Single	22	29.3
Educational level		
Diploma	14	18.7
Bachelor	46	61.3
Master	12	16.0
PhD	3	4.0
Current working place		
Inpatient departments	39	52.0
Outpatient clinics	36	48.0
Years of experience		
5–9	42	56.0
10 or more	33	44.0
Workshops (history taking)	32	42.7
Workshops (communication)	29	38.7
Problems with patients	8	10.7

Table 3 highlights notable advancements in nurses' knowledge across all evaluated areas following the educational program. The most substantial improvements were observed in the areas of communication, therapeutic relationships, psychiatric history-taking, emotional management and de-escalation, interdisciplinary collaboration, and the application of role-play and simulation as educational tools (all *p* < 0.001). Although cultural competence also improved, the increase was not as significant as in the other areas. Overall, knowledge levels transitioned significantly from mainly moderate before the intervention to adequate or highly adequate afterward, with no participants remaining in the moderate category (*χ*² = 123.046, *p* < 0.001). Supporting these results, the average total knowledge score rose markedly from 57.9 ± 5.1 before the intervention to 69.8 ± 3.9 afterward (*t* = 15.995, *p* < 0.001), demonstrating the effectiveness of the simulation-based educational program in improving psychiatric nurses' communication and history-taking skills and knowledge.

**Table 3:** Comparison of Pre- and post-intervention knowledge assessment in communication and history-taking competencies.

Domain	Pre-intervention (*n*, %)	Post-intervention (*n*, %)	Chi-square/Fisher’s exact test
Importance of effective communication	Disagree/Unsure: 68 (90.6%) Agree: 7 (9.3%)	Disagree/Unsure: 34 (45.3%) Agree: 41 (54.7%)	X² = 35.696, *p* < 0.001**
Building therapeutic relationships (rapport, empathy, trust)	Disagree/Unsure: 78 (83.9%) Agree: 15 (16.1%)	Disagree/Unsure: 57 (75.3%) Agree: 41 (54.7%)	X² = 16.684, *p* < 0.001**
Psychiatric history-taking skills	Disagree/Unsure: 77 (73.3%) Agree: 28 (26.7%)	Disagree/Unsure: 65 (42.0%) Agree: 90 (58.0%)	X² = 18.160, *p* < 0.001**
Managing challenging situations (emotional distress, de-escalation)	Disagree/Unsure: 85 (78.0%) Agree: 24 (22.0%)	Disagree/Unsure: 53 (45.3%) Agree: 64 (54.7%)	X² = 21.706, *p* < 0.001**
Cultural competence	Disagree/Unsure: 76 (58.7%) Agree: 53 (41.3%)	Disagree/Unsure: 58 (51.3%) Agree: 55 (48.7%)	X² = 16.436, *p* < 0.001**
Role-playing and simulation	Disagree/Unsure: 81 (72.0%) Agree: 32 (28.0%)	Disagree/Unsure: 59 (49.3%) Agree: 61 (50.7%)	X² = 8.090, *p* = 0.018*
Interdisciplinary collaboration	Disagree/Unsure: 75 (73.4%) Agree: 27 (26.6%)	Disagree/Unsure: 52 (36.7%) Agree: 89 (63.3%)	X² = 7.437, *p* = 0.024*
Total knowledge level	Moderate: 67 (89.3%) Adequate: 6 (8.0%) Highly Adequate: 2 (2.7%)	Moderate: 0 (0.0%) Adequate: 72 (96.0%) Highly Adequate: 3 (4.0%)	X² = 123.046, *p* < 0.001**
Mean score ± SD	57.9 ± 5.1	69.8 ± 3.9	t = 15.995, *p* < 0.001**

***p* < 0.05** indicates statistical significance; *p*** < 0.001** indicates highly significant differences. Categories are summarized by domain to simplify multiple items while retaining the statistical interpretation.

*meanly signficant; **means highly signficant.

Table 4 illustrates the evaluation of the effect size of educational interventions, highlighting their significant positive influence on physicians' knowledge and nursing skills compared to their initial levels before training. The educational intervention resulted in substantial to very substantial enhancements in knowledge, as evidenced by a large effect size (dᶻ = 1.85), indicating a marked increase in nurses' theoretical understanding. Additionally, communication skills saw notable improvement following participation in the educational program, with a large effect size (dᶻ = 0.99), signifying that the nurses' ability to develop and utilize interpersonal skills was enhanced after the educational session. The nurses who underwent training demonstrated a significant enhancement in their history-taking skills, with a large positive effect size (dᶻ = 3.20) on their capability to conduct thorough psychiatric assessments. Consequently, the findings advocate for the adoption of structured educational methods centered on competencies, such as history-taking skills, which are recognized as significantly advancing the development of these intricate clinical skills that psychiatric nurses have historically found challenging.

**Table 4:** Comparison of Pre- and post-intervention mean scores across knowledge, communication skills, and history-taking competencies (paired design).

Assessment tool	Pre-intervention (Mean ± SD)	Post-intervention (Mean ± SD)	*t*-value	*p*-value	Effect size (Cohen’s dᶻ)
Knowledge assessment scale	57.9 ± 5.1	69.8 ± 3.9	15.995	<0.001**	1.85
Communication skills checklist	54.0 ± 2.7	58.2 ± 3.3	8.522	<0.001**	0.99
History-taking competency	12.2 ± 4.4	32.3 ± 4.4	27.742	<0.001**	3.20

*p* < 0.001 indicates a highly significant improvement following the educational intervention. Cohen’s *d^z^* was calculated for paired samples using the formula *d^z^ = t/√I*. Effect size interpretation: 0.2 = small, 0.5 = moderate, 0.8 = large.

**highly significant.

**Figure 3:**
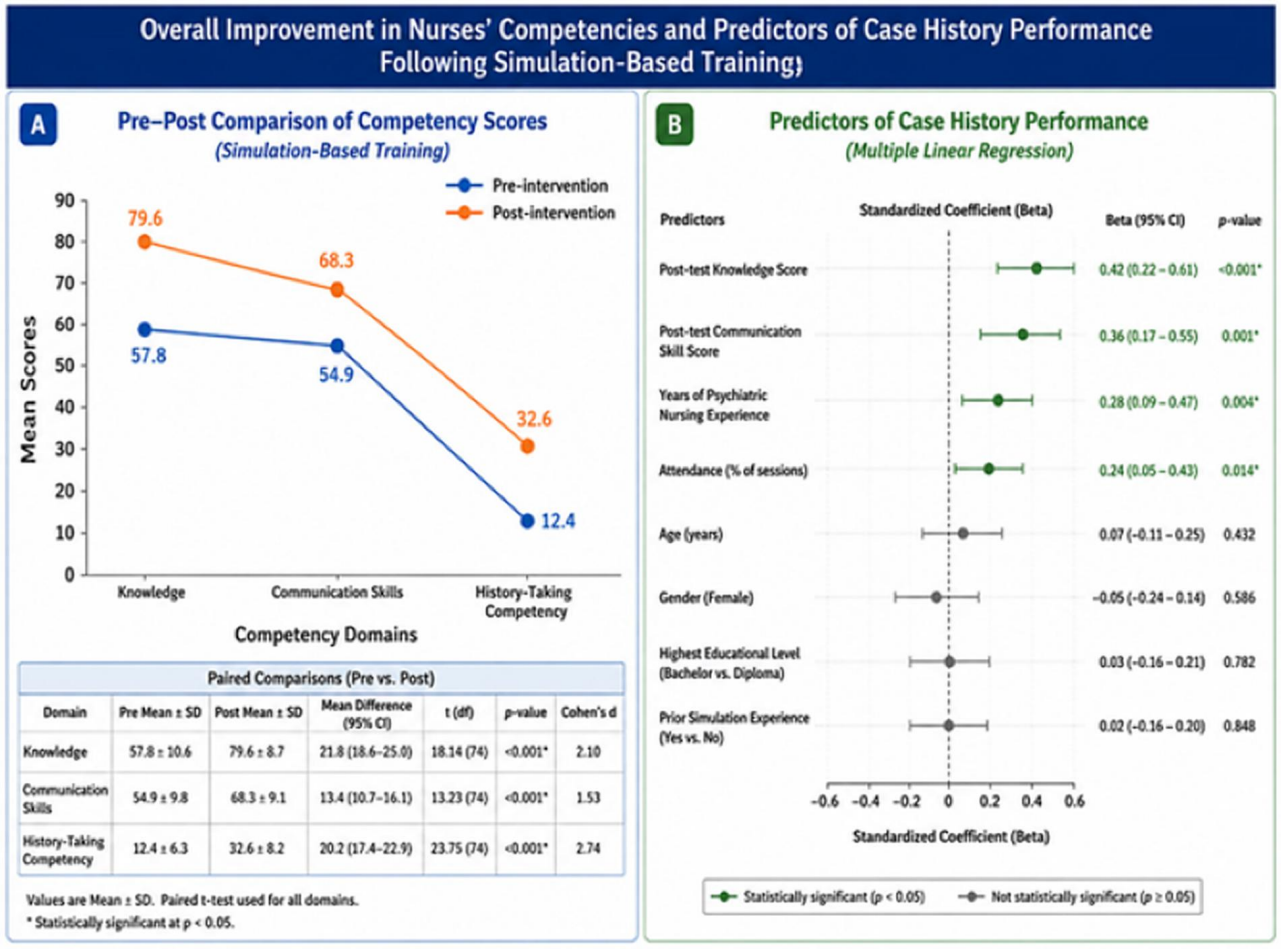
Overall improvement in nurses’ competencies and predictors of post-intervention performance across knowledge, communication skills, and history-taking competencies.

Figure 3 demonstrates notable enhancements in the participants' results after engaging in the simulation-based educational program. The most significant improvement was observed in history-taking abilities, with subsequent gains in knowledge and communication skills. Each enhancement was statistically significant and linked to large effect sizes, highlighting the strong positive influence of the intervention. Furthermore, after the intervention, history-taking performance was significantly correlated with higher knowledge scores, improved communication skills, more extensive psychiatric nursing experience, and increased attendance rates. Conversely, age, sex, educational level, and prior simulation training did not significantly correlate with post-intervention performance. These results imply that active involvement in the program and better communication skills enhance history-taking performance.

Table 5 indicates that the checklist assessing communication skills based on direct observation showed significant improvement in all categories after the educational intervention. Before the intervention, none of the 48 nurses sampled demonstrated full competency in their ability to communicate effectively. After completing the course, 48.0% of the sample exhibited competency, while 52.0% displayed partial competency. Furthermore, all individual categories (greeting the patient, establishing eye contact, utilizing language effectively, using active listening skills, and expressing empathy) displayed statistically significant improvements at *p* < 0.001 following the completion of the intervention. The mean score increased from 9.2 ± 3.2 to 32.8 ± 3.4, indicating that the educational intervention resulted in a statistically significant increase in the Psychiatric Healthcare communication skills of the nurses in the sample group.

**Table 5:** Comparison of the pre and post observation-based communication skill checklist *N* = 75.

Items of observation	Pre		Post		Chi-square/Fisher’s exact test	
	*n*	%	*n*	%	X^2^	*P*
Greets the patient	19	25.3	55	73.3	34.566	<0.001**
Check the patient’s readiness for communication through verbal (agree) and nonverbal facial expressions (smile, and head nodding) and take his permission to start interaction.	14	18.7	45	60.0	26.849	<0.001**
Introduces self by name and category	14	18.7	56	74.7	47.250	<0.001**
Sits squarely facing the client.	16	21.3	53	70.7	36.742	<0.001**
Maintains open relaxed erect posture	15	20.0	50	66.7	33.258	<0.001**
Has neutral facial expression (mild anxiety is acceptable)	9	12.0	63	84.0	77.885	<0.001**
Leans forward towards the client	14	18.7	59	78.7	54.038	<0.001**
Establishes eye contact while demonstrating cultural sensitivity.	14	18.7	58	77.3	51.709	<0.001**
Maintains personal space (not too close or away from the patient)	14	18.7	55	73.3	45.115	<0.001**
Avoids (social comments/behaviors e.g., we will be friends, indiscriminative use of touch)	16	21.3	54	72.0	38.679	<0.001**
Neat appearance and wears a white apron.	20	26.7	56	74.7	34.566	<0.001**
Ensures the comfort of patients in the environment.	13	17.3	56	74.7	49.624	<0.001**
Shows respect for the patient’s privacy & confidentiality.	19	25.3	55	73.3	34.566	<0.001**
Shows respect for the patient’s culture, and beliefs.	18	24.0	52	69.3	30.964	<0.001**
Establish trust by non -threatening & non-judgmental behavior	19	25.3	52	69.3	29.123	<0.001**
Addresses the patient by name.	17	22.7	58	77.3	44.827	<0.001**
Ask for a reason to visit.	13	17.3	51	68.0	39.353	<0.001**
Provide an overview of the interview’s purpose.	14	18.7	51	68.0	37.167	<0.001**
Uses everyday language.	17	22.7	56	74.7	40.589	<0.001**
Uses appropriate vocabulary.	18	24.0	58	77.3	42.674	<0.001**
Explains medical terms (if used).	18	24.0	62	82.7	51.857	<0.001**
Matches voice tone and intonation with the verbal content.	14	18.7	56	74.7	47.250	<0.001**
Active listening with minimal interruption.	15	20.0	61	81.3	56.437	<0.001**
Avoids self-touching and non-purposive movements.	11	14.7	57	76.0	56.923	<0.001**
Frequent vertical head nods.	19	25.3	55	73.3	34.566	<0.001**
Occasional Vocalization	12	16.0	59	78.7	59.075	<0.001**
Appropriate length of eye contact is comfortable to the patient	12	16.0	56	74.7	52.080	<0.001**
Forward lean facing the patient.	10	13.3	42	56.0	30.141	<0.001**
Open posture with uncrossed arms.	13	17.3	52	69.3	41.294	<0.001**
Matches facial expressions with verbal content.	15	20.0	61	81.3	56.437	<0.001**
Allow the patient to narrate illness.	19	25.3	47	62.7	21.212	<0.001**
Asks open-ended questions (avoids double barrel & negative questions).	14	18.7	60	80.0	56.437	<0.001**
Allow wait time for patients to respond.	19	25.3	47	62.7	21.212	<0.001**
Probe understanding and reinstate important points.	15	20.0	58	77.3	49.342	<0.001**
Checks accuracy of the statement by getting the patient’s approval.	16	21.3	55	73.3	40.676	<0.001**
Responds to the patient’s emotions.	12	16.0	54	72.0	47.727	<0.001**
Demonstrate understanding of a patient’s rationale for behavior or illness.	19	25.3	55	73.3	34.566	<0.001**
Appreciates the patient’s coping efforts.	18	24.0	51	68.0	29.227	<0.001**
Offers supportive words of assurance.	13	17.3	59	78.7	56.517	<0.001**
Express willingness to help	13	17.3	50	66.7	37.466	<0.001**
Express understanding of patients’ barriers to compliance.	13	17.3	57	76.0	51.857	<0.001**
Express concern for patients.	16	21.3	53	70.7	36.742	<0.001**
Express understanding of patient beliefs.	12	16.0	56	74.7	52.080	<0.001**
Asks patients for any unresolved issues.	18	24.0	56	74.7	38.514	<0.001**
Provide guidance on further action plan	18	24.0	52	69.3	30.964	<0.001**
Total checklist level	*n*	%	*n*	%	X^2^	*P*
Not competent	75	100.0	0	0.0		
Partially competent	0	0.0	39	52.0		
Competent	0	0.0	36	48.0	150.000	<0.001**
Mean ± SD	9.2 ± 3.2	32.8 ± 3.4	44.053	<0.001**		

*meanly significant; **means highly significant.

Table 6 demonstrates significant improvements in nurses' psychiatric history-taking competencies following the educational intervention. The total competency score increased from 12.21 ± 4.44 pre-intervention to 32.33 ± 4.44 post-intervention (*p* < 0.001), with a very large overall effect size (Cohen's d = 4.53). Significant improvements were observed across all domains of psychiatric history-taking competence (*p* < 0.001), with large effect sizes ranging from d = 1.07 to 1.25. The greatest improvements were noted in comprehensive psychiatric history-taking, therapeutic communication, professionalism during assessment, and mental status examinations. Overall, these findings indicate that the educational intervention produced substantial and clinically meaningful improvements in psychiatric history-taking competencies among the participating nurses.

**Table 6:** Nurses’ psychiatric history-taking competency before and after the educational intervention (*N* = 75).

Psychiatric history-taking competency domains	Pre-intervention mean ± SD	Post-intervention mean ± SD	Mean difference	*p*-value	Effect size (Cohen’s d)
1. Establishes therapeutic rapport with patient	0.41 ± 0.49	0.88 ± 0.32	+0.47	<0.001	1.15
2. Introduces self and explains purpose of interview	0.39 ± 0.49	0.86 ± 0.35	+0.47	<0.001	1.10
3. Obtains chief complaint appropriately	0.44 ± 0.50	0.91 ± 0.28	+0.47	<0.001	1.18
4. Assesses history of present illness comprehensively	0.36 ± 0.48	0.84 ± 0.37	+0.48	<0.001	1.12
5. Explores onset and duration of symptoms	0.38 ± 0.49	0.87 ± 0.34	+0.49	<0.001	1.15
6. Assesses severity and progression of symptoms	0.35 ± 0.48	0.85 ± 0.36	+0.50	<0.001	1.17
7. Obtains past psychiatric history accurately	0.33 ± 0.47	0.82 ± 0.39	+0.49	<0.001	1.10
8. Assesses history of psychiatric hospitalization	0.31 ± 0.46	0.80 ± 0.40	+0.49	<0.001	1.08
9. Assesses medication history effectively	0.42 ± 0.50	0.89 ± 0.31	+0.47	<0.001	1.16
10. Explores family psychiatric history	0.34 ± 0.47	0.81 ± 0.39	+0.47	<0.001	1.07
11. Assesses family support system	0.45 ± 0.50	0.90 ± 0.30	+0.45	<0.001	1.10
12. Obtains personal and social history appropriately	0.37 ± 0.48	0.86 ± 0.35	+0.49	<0.001	1.14
13. Assesses educational and occupational history	0.40 ± 0.49	0.88 ± 0.32	+0.48	<0.001	1.15
14. Explores substance use history	0.29 ± 0.45	0.79 ± 0.41	+0.50	<0.001	1.12
15. Assesses medical and surgical history	0.41 ± 0.49	0.87 ± 0.34	+0.46	<0.001	1.11
16. Performs mental status examination appropriately	0.32 ± 0.47	0.84 ± 0.37	+0.52	<0.001	1.20
17. Assesses patient mood and affect	0.35 ± 0.48	0.86 ± 0.35	+0.51	<0.001	1.18
18. Assesses thought process and content	0.30 ± 0.46	0.82 ± 0.39	+0.52	<0.001	1.16
19. Evaluates perception disturbances	0.28 ± 0.45	0.80 ± 0.40	+0.52	<0.001	1.15
20. Assesses cognitive functioning	0.36 ± 0.48	0.85 ± 0.36	+0.49	<0.001	1.13
21. Assesses insight and judgment	0.33 ± 0.47	0.83 ± 0.38	+0.50	<0.001	1.14
22. Evaluates suicidal ideation and risk	0.27 ± 0.44	0.78 ± 0.42	+0.51	<0.001	1.11
23. Evaluates homicidal ideation and risk	0.25 ± 0.43	0.77 ± 0.42	+0.52	<0.001	1.12
24. Demonstrates effective documentation skills	0.39 ± 0.49	0.89 ± 0.31	+0.50	<0.001	1.19
25. Demonstrates organized interview sequence	0.41 ± 0.49	0.90 ± 0.30	+0.49	<0.001	1.18
26. Demonstrates therapeutic communication throughout interview	0.43 ± 0.50	0.92 ± 0.27	+0.49	<0.001	1.21
27. Summarizes findings appropriately	0.37 ± 0.48	0.86 ± 0.35	+0.49	<0.001	1.14
28. Identifies patient priority problems	0.34 ± 0.47	0.84 ± 0.37	+0.50	<0.001	1.15
29. Demonstrates professionalism during assessment	0.45 ± 0.50	0.93 ± 0.25	+0.48	<0.001	1.20
30. Completes psychiatric history-taking comprehensively	0.38 ± 0.49	0.91 ± 0.28	+0.53	<0.001	1.25
Overall Psychiatric History-Taking Competency Score	12.21 ± 4.44	32.33 ± 4.44	+20.12	<0.001	4.53

Checklist scoring: 0 = Not Done, 1 = Done. Higher scores indicate better psychiatric history-taking competency.

Effect size interpretation: Small = 0.20–0.49, Moderate = 0.50–0.79, Large ≥ 0.80.

Table 7 presents the association between nurses' demographic characteristics and their post-intervention knowledge levels. No significant associations were found between age, sex, workplace, participation in history-taking workshops, or reported patient-related problems (*p* > 0.05). However, the knowledge level was significantly associated with marital status (*χ*² = 7.217, *p* = 0.027), educational level (*χ*² = 25.586, *p* < 0.001), and years of experience (*χ*² = 11.949, *p* = 0.003). Married nurses, those with higher educational qualifications, and those with ≥10 years of experience were more likely to achieve adequate or highly adequate knowledge levels following the intervention. Attendance at communication workshops showed a borderline association with knowledge level (*p* = 0.050). Overall, educational attainment and professional experience emerged as the strongest factors associated with postintervention knowledge outcomes.

**Table 7:** Association between the demographic characteristics of the study sample and knowledge assessment scale level (post-intervention).

	Moderate knowledge		Adequate knowledge		Highly adequate knowledge		Chi – square/Fisher’s exact test	
	*n*	%	*n*	%	*n*	%	X^2^	*P*
Age (Years)								
25–35	3	50.0	25	52.1	7	33.3		
36–46	2	33.3	15	31.3	10	47.6		
>46	1	16.7	8	16.7	4	19.0	2.266	0.687
Gender								
Female	3	50.0	24	50.0	13	61.9		
Male	3	50.0	24	50.0	8	38.1	0.861	0.650
Marital status								
Married	6	100.0	29	60.4	18	85.7		
Single	0	0.0	19	39.6	3	14.3	7.217	0.027*
Educational level								
Diploma	3	50.0	11	22.9	0	0.0		
Bachelor	3	50.0	33	68.8	10	47.6		
Master	0	0.0	4	8.3	8	38.1		
PhD	0	0.0	0	0.0	3	14.3	25.586	<0.001**
Current working place								
Inpatient departments	3	50.0	26	54.2	10	47.6		
Outpatient clinics	3	50.0	22	45.8	11	52.4	0.261	0.877
Years of experience								
5–9	6	100.0	30	62.5	6	28.6		
10 or more	0	0.0	18	37.5	15	71.4	11.949	0.003*
Workshops (history taking)	3	50.0	18	37.5	11	52.4	1.466	0.481
Workshops (communication)	5	83.3	18	37.5	6	28.6	5.978	0.050
Problems with patients	0	0.0	7	14.6	1	4.8	2.258	0.323

**means highly signficant.

Table 8 presents the association between nurses' demographic characteristics and post-intervention communication competency. No significant associations were found between age, gender, marital status, workplace, previous participation in history-taking or communication workshops, or reported patient-related problems (*p* > 0.05). However, communication competency was significantly associated with educational level (*χ*² = 30.496, *p* < 0.001) and years of experience (*χ*² = 12.304, *p* = 0.002) in this study. Nurses with bachelor's, master's, or doctoral degrees were more likely to demonstrate communication competency than diploma-prepared nurses, while those with 10 or more years of experience were more likely to achieve full competency than those with 5–9 years of experience.

**Table 8:** Association between the demographic characteristics of the study sample and observation-based communication skill checklist level (post-intervention).

	Not competent		Partially competent		Competent		Chi-square/Fisher’s exact test	
	*n*	%	*n*	%	*n*	%	X^2^	*P*
Age (Years)								
25–35	2	50.0	17	48.6	16	44.4		
36–46	1	25.0	11	31.4	15	41.7		
>46	1	25.0	7	20.0	5	13.9	1.259	0.868
Gender								
Female	4	100.0	19	54.3	17	47.2		
Male	0	0.0	16	45.7	19	52.8	4.053	0.132
Marital status								
Married	2	50.0	24	68.6	27	75.0		
Single	2	50.0	11	31.4	9	25.0	1.224	0.542
Educational level								
Diploma	4	100.0	10	28.6	0	0.0		
Bachelor	0	0.0	19	54.3	27	75.0		
Master	0	0.0	6	17.1	6	16.7		
PhD	0	0.0	0	0.0	3	8.3	30.496	<0.001**
Current working place								
Inpatient departments	2	50.0	21	60.0	16	44.4		
Outpatient clinics	2	50.0	14	40.0	20	55.6	1.727	0.422
Years of experience								
5–9	4	100.0	25	71.4	13	36.1		
10 or more	0	0.0	10	28.6	23	63.9	12.304	0.002*
Workshops (history taking)	2	50.0	13	37.1	17	47.2	0.830	0.660
Workshops (communication)	3	75.0	14	40.0	12	33.3	2.685	0.261
Problems with patients	1	25.0	2	5.7	5	13.9	2.156	0.340

*meanly significant.

Table 9 shows that post-intervention case history competency was not significantly associated with age, sex, marital status, workplace, or previous participation in communication and history-taking workshops (*p* > 0.05). However, significant associations were observed for educational level (*χ*² = 40.722, *p* < 0.001), years of experience (*χ*² = 12.429, *p* = 0.002), and patient-related problems (*χ*² = 9.006, *p* = 0.011). Nurses with higher educational qualifications, greater psychiatric nursing experience, and fewer patient-related problems were more likely to achieve higher levels of case history competency after the intervention.

**Table 9:** Association between the demographic characteristics of the study sample and nurses’ case history level (post – intervention).

	Not competent		Partially competent		Competent		Chi-square/Fisher’s exact test	
	*n*	%	*n*	%	*n*	%	X^2^	*P*
Age (Years)								
25–35	5	45.5	11	52.4	19	44.2		
36–46	2	18.2	7	33.3	18	41.9		
>46	4	36.4	3	14.3	6	14.0	4.323	0.364
Gender								
Female	7	63.6	14	66.7	19	44.2		
Male	4	36.4	7	33.3	24	55.8	3.415	0.181
Marital status								
Married	8	72.7	16	76.2	29	67.4		
Single	3	27.3	5	23.8	14	32.6	0.547	0.761
Educational level								
Diploma	9	81.8	5	23.8	0	0.0		
Bachelor	2	18.2	12	57.1	32	74.4		
Master	0	0.0	4	19.0	8	18.6		
PhD	0	0.0	0	0.0	3	7.0	40.722	<0.001**
Current working place								
Inpatient departments	5	45.5	13	61.9	21	48.8		
Outpatient clinics	6	54.5	8	38.1	22	51.2	1.187	0.553
Years of experience								
5–9	11	100.0	13	61.9	18	41.9		
10 or more	0	0.0	8	38.1	25	58.1	12.429	0.002*
Workshops (history taking)	5	45.5	6	28.6	21	48.8	2.410	0.300
Workshops (Communication)	3	27.3	9	42.9	17	39.5	0.771	0.680
Problems with patients	4	36.4	1	4.8	3	7.0	9.006	0.011*

*meanly significant; **means highly significant.

**Figure 4:**
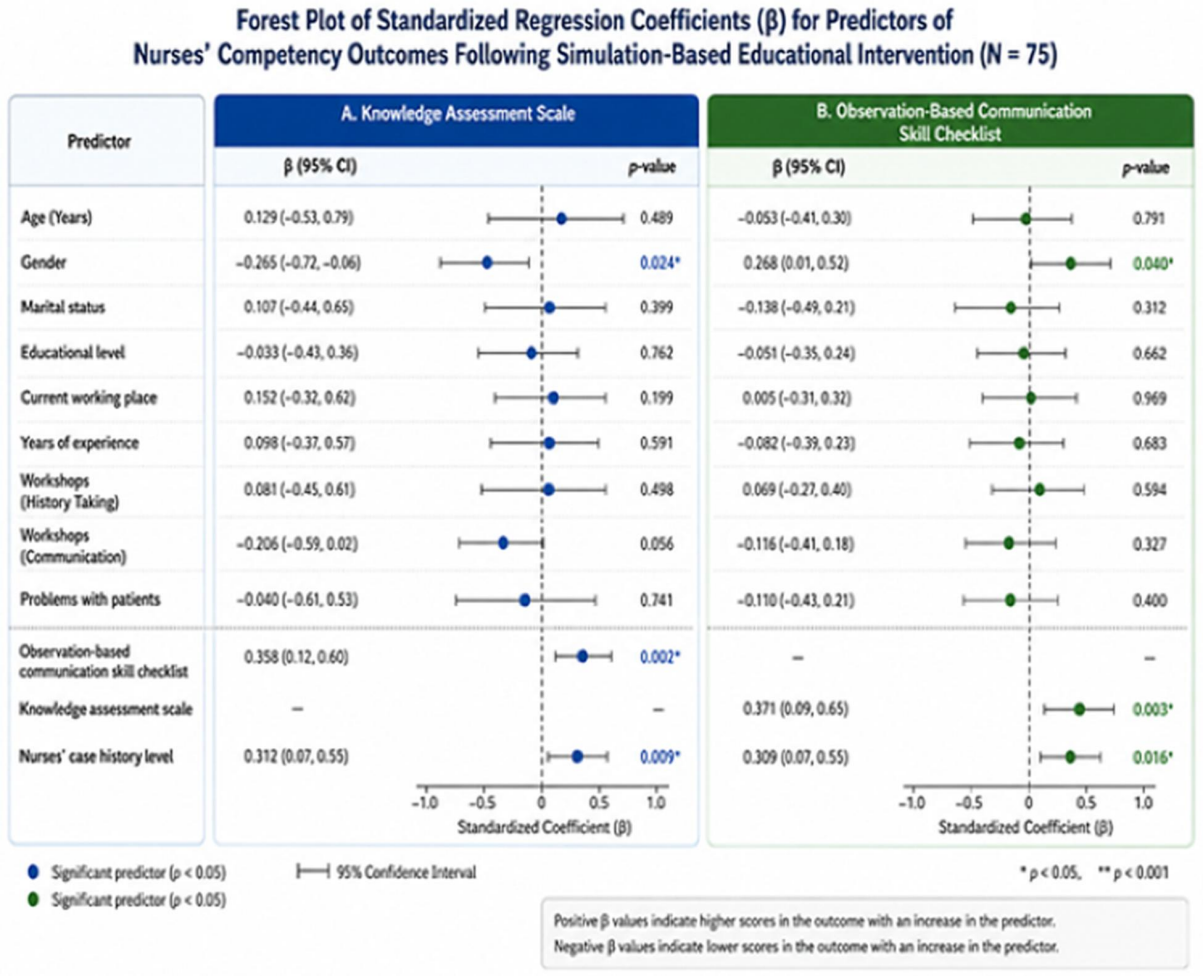
Forest plot of standardized regression coefficients (*β*) for predictors of nurses’ competency outcomes following simulation-based educational intervention (*N* = 75).

Figure 4 presents the standardized regression coefficients (*β*) and 95% confidence intervals for the predictors of post-intervention knowledge and communication competency. For knowledge outcomes (Panel A), communication competency (*β* = 0.358, *p* = 0.002) and psychiatric history-taking competency (*β* = 0.312, *p* = 0.009) were significant positive predictors, whereas sex showed a significant negative association (*β* = −0.265, *p* = 0.024). For communication competency outcomes (Panel B), knowledge scores (*β* = 0.371, *p* = 0.003), psychiatric history-taking competency (*β* = 0.309, *p* = 0.016), and gender (*β* = 0.268, *p* = 0.040) were significant predictors of communication competency outcomes. Most demographic and occupational variables were not significantly associated with either of the outcomes. Overall, knowledge, communication skills, and psychiatric history-taking competency were the strongest predictors of post-intervention performance.

## Discussion

The results of this study align with the increasing evidence that educational interventions using simulations are effective in improving psychiatric nurses' skills in therapeutic communication and psychiatric assessment. Dawood et al. (26) similarly found that using standardized patient simulation greatly enhanced communication abilities, empathy, confidence, and therapeutic engagement in psychiatric and mental health nursing students. In a related study, Liu et al. (27) observed that simulation-based training in therapeutic communication boosted nursing students' competence in communication, self-efficacy, and confidence during patient interactions. Cabañero-Martínez et al. (28) also reported that standardized patient simulation improved empathy, active listening, patient-centered communication, and therapeutic interaction skills among nursing students. The alignment between these studies and the current findings can be attributed to the experiential nature of simulation-based learning, which allows repeated practice, reflective learning, and immediate feedback in realistic psychiatric scenarios.

In contrast to conventional didactic teaching methods, simulation-based education allows psychiatric nurses to actively participate in realistic therapeutic interactions and practice their psychiatric interviewing skills in psychologically safe learning settings. Gutierrez-Puertas et al. (29) similarly observed that simulation-based psychiatric education enhances communication abilities and clinical confidence through experiential learning and repeated exposure to realistic mental health scenarios. These findings are further corroborated by Jeffries' simulation theory (30) and Kolb's experiential learning theory (22), both of which highlight the significance of active involvement, reflective observation, and integrating theoretical knowledge into clinical practice. Consequently, the use of structured psychiatric scenarios in the current study may have played a role in enhancing therapeutic engagement, communication fluency, and psychiatric assessment skills among the participating nurses.

The current results align with earlier research, indicating that communication competence encompasses more than just theoretical understanding; it also involves behavioral execution, contextual suitability, interpersonal sensitivity, and therapeutic interaction. Cant and Cooper (31) found that simulation-based learning enhances communication skills through repeated practice in secure clinical settings. Likewise, Cleary et al. (32) determined that standardized patient simulation offers more genuine and clinically pertinent communication experiences than traditional classroom instruction. The resemblance between these findings and those of the present study may highlight the effectiveness of psychiatric simulation training in alleviating communication anxiety and boosting nurses' confidence in handling emotionally delicate psychiatric scenarios.

The enhancement in psychiatric history-taking skills aligns with earlier studies that have highlighted the success of structured psychiatric simulation exercises in boosting psychiatric interviewing abilities. Siemerkus et al. (33) found that simulations with standardized psychiatric patients enhance performance in psychiatric assessments, especially in delicate areas such as suicidal thoughts, psychosocial stressors, trauma exposure, and substance use evaluation. Similarly, Wheeler and Delaney (34) pointed out that taking psychiatric histories involves the integration of therapeutic communication, psychosocial evaluation, clinical reasoning, and systematic information collection, all of which can be improved through practical psychiatric training. The resemblance between these findings and the current study might be due to the use of realistic psychiatric scenarios that allowed nurses to practice comprehensive psychiatric assessment skills in supportive and controlled learning environments.

Nevertheless, some earlier studies have presented findings that somewhat diverge from the current outcomes. For instance, Foronda et al. (35) observed that while simulations enhance confidence in communication and learner satisfaction, the long-term application of communication skills in everyday clinical settings might be inconsistent without ongoing reinforcement and mentorship. Similarly, Happell et al. (36) pointed out that factors such as organizational workload, staff shortages, lack of adequate psychiatric supervision, and workplace pressures can hinder the sustainability of communication improvements, even after successful educational interventions. These partially differing findings might clarify why sustaining communication competency in psychiatric environments often necessitates continuous reinforcement, reflective practice, and institutional backing beyond short-term educational initiatives to be effective.

The findings of the current study may also reflect the common challenges encountered in psychiatric nursing practice. Previous psychiatric nursing studies have demonstrated that insufficient psychiatric training, organizational barriers, workload pressures, and limited supervision negatively affect therapeutic nurse–patient communication and psychiatric interviewing practices (36). In the Saudi Arabian context, Alzahrani et al. (37) and Koenig et al. (38) reported that sociocultural stigma toward mental illness, hierarchical healthcare structures, and role ambiguity among psychiatric nurses may further contribute to reduced confidence and limited therapeutic communication practices in psychiatric settings. Therefore, the positive outcomes observed following the intervention may be partially explained by the structured opportunities provided for nurses to practice therapeutic communication and psychiatric assessment skills within supportive simulation environments, which may not always be available in routine psychiatric clinical practice (39–43).

The present findings also suggest that experiential learning strategies may exert a stronger influence on competency development than demographic or professional characteristics alone. Similar observations were reported by Yadeta et al. (44), who found that simulation-based educational strategies improved communication and assessment competencies regardless of years of experience or background characteristics. This finding reinforces the value of structured psychiatric simulation training as a standardized competency development strategy for psychiatric nurses (45–48).

Although the intervention demonstrated positive outcomes, sustaining communication and psychiatric interviewing competencies in routine practice may require ongoing reinforcement, mentorship, supervision, and continuous professional development. Lateef (49) reported that communication and interviewing skills acquired through simulation may decline over time without continuous clinical reinforcement and reflective practice opportunities. Consequently, integrating structured psychiatric simulation training into undergraduate nursing curricula, orientation programs, and continuing professional development initiatives may help maintain long-term competency improvements among psychiatric nurses (30, 33, 37, 40, 50, 51).

The findings of this study have important implications for psychiatric nursing education and clinical practice (43, 44, 47). Strengthening therapeutic communication and psychiatric assessment competencies through simulation-based psychiatric education may contribute to improved therapeutic relationships, more accurate psychiatric assessments, enhanced patient-centered care and better mental health outcomes.

## Limitations

This study has several limitations that should be considered when interpreting the findings. First, it was conducted in a single psychiatric hospital using a convenience sample composed primarily of Saudi nurses, which may limit the generalizability of the findings to other settings and populations. Although nurses from all rotating shifts were eligible to participate, training sessions were conducted during morning hours for logistical reasons, potentially introducing selection bias. Second, not all participants were licensed psychiatric nurses, and variations in participant engagement may have influenced the application of acquired skills. Third, the quasi-experimental design without a control group limits causal inference. Finally, outcomes were assessed immediately following the intervention, and neither long-term retention of competencies nor patient-related and real-world clinical outcomes were evaluated.

## Conclusion

The results of this quasi-experimental study suggest that simulation-based clinical training may be an effective method for enhancing nurses' communication skills and their ability to collect patient histories in psychiatric settings. By conceptualizing nurse–patient interactions as structured, therapeutic, and patient-focused processes, the intervention led to statistically significant enhancements in nurses' knowledge and self-assessed skills related to clinical communication. These findings underscore the importance of experiential learning, active engagement, and reflective practice in facilitating the application of theoretical knowledge to clinical settings.

Nevertheless, the consistent use of these skills in real-world scenarios may be affected by organizational, professional, and contextual factors that were not addressed in this study. Despite these considerations, simulation-based training emerges as a promising approach for advancing psychiatric nursing education and promoting recovery-oriented care. Further research employing randomized designs and extended follow-up periods is advised to assess the sustainability and generalizability of these outcomes.

## Recommendations

Training based on simulations should be incorporated into psychiatric nursing education and ongoing professional development initiatives to enhance communication and history-taking skills. Healthcare organizations must support these initiatives by providing sufficient resources, mentorship, oversight, and reinforcement strategies that enable the transfer and long-term application of learned skills in clinical practice. Well-defined roles, competency models, and systematic performance appraisal processes can enhance the preservation of therapeutic communication skills in psychiatric nurses. Future studies should utilize multicenter and longitudinal designs to evaluate the longevity of training impacts, improve the applicability of results, and investigate the effects of organizational and personal factors on competency retention. Moreover, integrating blended and digital simulation methods and assessing patient-centered results, such as therapeutic engagement, patient-reported outcomes, and quality of care metrics, would provide broader insights into the clinical effects of simulation-based educational interventions.

## Data Availability

The original contributions presented in the study are included in the article/Supplementary Material, further inquiries can be directed to the corresponding author.

## Supplementary material

The Supplementary Material for this article can be found online at: https://www.frontiersin.org/articles/10.3389/frhs. 2026.1847509/full#supplementary-material
