# Supplementary material for: Simulation-based training for enhancing psychiatric nurses’ knowledge and clinical competencies in communication and psychiatric history taking: a quasi-experimental study
Source: Front Health Serv. 2026 Jul 22;6:1847509. doi: 10.3389/frhs.2026.1847509 (PMC13437964; doi:10.3389/frhs.2026.1847509)
Supplement: Supplementary file 1 [file Datasheet1.pdf]

Knowledge assessment scale (khalil A., 2025)

No.	statements	Agree	unsure	Disagree
1.	Effective communication is crucial in psychiatric nursing.			
2.	Managing emotional distress is a unique challenge when communicating with psychiatric patients.			
3.	Demonstrating empathy and active listening is a key technique for establishing rapport and trust in psychiatric nursing			
4.	Maintaining a non-judgmental attitude fosters trust and openness in building a therapeutic relationship.			
5.	Establishing rapport and trust is an ongoing process in psychiatric nursing.			
6.	Using therapeutic communication techniques is essential for developing a positive nurse-patient relationship.			
7.	Nurses should actively involve patients in their care decisions to strengthen the therapeutic relationship.			
8.	Body language is an example of a non-verbal communication technique.			
9.	Empathetic understanding and reflection are key components of active listening skills.			
10.	Empathetic responding and validation techniques aim to acknowledge and understand the patient's feelings.			
11.	A comprehensive psychiatric history provides insight into the patient's mental health.			
12.	Following a systematic set of relevant questions and prompts is a structured approach to psychiatric history taking.			
13.	Asking open-ended questions during history taking encourages patients to share more about their experiences.			
14.	Allowing patients to share their narrative without interruption is crucial for gathering a comprehensive psychiatric history.			
15.	Collaborating with family members or caregivers is valuable in obtaining a thorough psychiatric history			
16.	Considering cultural and linguistic factors is important when conducting psychiatric history taking.			
17.	Regularly reviewing and updating the psychiatric history is necessary for effective patient care.			
18.	Exploring the patient's concerns and feelings is a strategy for handling resistance or emotional distress during communication			
19.	De-escalation techniques aim to calm and reduce agitation in the patient.			
20.	You feel uncomfortable on the unit during emotionally charged situations with Psychiatric patients.			
21.	Addressing patient resistance during history-taking is a part of effective communication			
22.	Demonstrating empathy during challenging situations can positively affect the nurse-patient relationship.			
23.	Collaborating with the interdisciplinary team is valuable in managing challenging situations during patient interactions.			

24.	Avoid discussing sensitive topics during history taking during patient’s interaction			
25.	Understanding cultural nuances is crucial for providing culturally sensitive care.			
26.	Adapting care to respect and meet individual cultural needs ensures culturally sensitive care.			
27.	Practical scenarios for applying communication skills provide opportunities for continuous improvement.			
28.	Feedback sessions are important to identify mistakes and areas for improvement in role-playing and simulation exercises.			
29.	Collaborating with other healthcare professionals ensures a comprehensive and coordinated care plan for holistic patient care.			
30.	Promoting open and effective communication enhances communication within the healthcare team.			
